# Measures of family planning service quality associated with contraceptive discontinuation: an analysis of Measurement, Learning & Evaluation (MLE) project data from urban Kenya

**DOI:** 10.12688/gatesopenres.12974.2

**Published:** 2020-01-29

**Authors:** Karla Feeser, Nirali M. Chakraborty, Lisa Calhoun, Ilene S. Speizer

**Affiliations:** 1 Metrics for Management, Baltimore, MD, 21201, USA; 2 Carolina Population Center, University of North Carolina at Chapel Hill, Chapel Hill, NC, 27516, USA

**Keywords:** Contraceptive discontinuation, Family planning, quality, measurement, Kenya

## Abstract

**Introduction: **Several measures to assess family planning service quality (FPQ) exist, yet there is limited evidence on their association with contraceptive discontinuation. Using data from the Measurement, Learning & Evaluation (MLE) Project, this study investigates the association between FPQ and discontinuation-while-in-need without switching in five cities in Kenya. Two measures of FPQ are examined – the Method Information Index (MII) and a comprehensive service delivery point (SDP) assessment rooted in the Bruce Framework for FPQ.

**Methods: **Three models were constructed: two to assess MII reported in household interviews (as an ordinal and binary variable) among 1,033 FP users, and one for facility-level quality domains among 938 FP users who could be linked to a facility type included in the SDP assessment. Cox proportional hazards ratios were estimated where the event of interest was discontinuation-while-in-need without switching. Facility-level FPQ domains were identified using exploratory factor analysis (EFA) using SDP assessment data from 124 facilities.

**Results: **A woman’s likelihood of discontinuation-while-in-need was approximately halved whether she was informed of one aspect of MII (HR: 0.45, p < 0.05), or all three (HR: 0.51, p < 0.01) versus receiving no information, when MII was assessed as an ordinal variable. Six facility-level quality domains were identified in EFA. Higher scores in information exchange, privacy, autonomy & dignity and technical competence were associated with a reduced risk of discontinuation-while-in-need (p < 0.05).

**Conclusions: **The MII has potential as an actionable metric for FPQ monitoring at the health facility level. Furthermore, family planning facilities and programs should emphasize information provision and client-centered approaches to care alongside technical competence in the provision of FP care.

## Introduction

Despite global advances in access to family planning services, 214 million women of reproductive age in developing regions of the world still experience an unmet need for modern contraception ^1^. Contraceptive discontinuation is understood to be a driver of unmet need for family planning; in fact, an analysis of Demographic and Health Survey (DHS) data collected in 34 countries between 2005 and 2010 demonstrated that modern method discontinuation while in need—which occurs when a woman who wishes to avoid pregnancy stops using her modern method of contraception—accounted for over one third total estimated unmet need ^2^. When a person is able to continue use of their contraceptive method for the duration of their time in need, it may be an indication that the health system has met their need for family planning care. Furthermore, there is evidence to suggest that the quality of family planning (FP) services can impact continued contraceptive use ^3– 6^. Thus, contraceptive discontinuation provides a key measurable outcome of interest in quality improvement strategies in FP service provision; however, identifying which aspects of structural and process quality are correlated with contraceptive method discontinuation can provide insights that are actionable for health systems implementers now—before poor quality service delivery manifests as unmet need.

The assessment of quality of care (QoC) in family planning programs has been largely guided by the Bruce family planning QoC framework for the past several decades. The framework articulates six fundamental domains of quality: choice of methods, information given to clients, technical competence of providers, interpersonal relations, mechanisms for follow-up and having an appropriate constellation of services ^7^. A number of measurement tools exist which include indicators that seek to capture key elements of FP service quality; yet, questions remain as to the utility of existing tools for performance benchmarking and strategic decision making ^8^. The broadly endorsed Bruce framework is applied inconsistently among researchers and programs seeking to understand and improve quality of care at the facility level, and limited guidance exists as to how to analyze resulting data once collected. A recent review of quality assessment tools for FP programs in LMICs identified 20 comprehensive tools for the assessment of clinical quality of care ^9^. Some are well aligned with the Bruce framework. For example, the Quick Investigation of Quality (QIQ) was developed by MEASURE Evaluation in 2000 as a tool that would balance the feasibility of data collection with the reliability of the resulting data. The QIQ indicators align with 5 out of the 6 domains of FP service quality defined in the Bruce Framework: choice of methods, information provision, technical competence, interpersonal relations, and mechanisms for follow-up ^10^. Meanwhile, large scale facility surveys, such as the Service Availability and Readiness Assessment (SARA) used by WHO for health facility assessment in LMICs, typically limit data collection to facility audits which assess infrastructure and readiness for choice only ^11^. Indeed, these structural aspects of quality are among the easiest and least expensive to measure.

When data to measure the domains of the Bruce framework are collected inconsistently, it creates an analytical challenge for programs that wish to summarize and compare findings related to the measurement of FP QoC. Some programs have tracked changes over time in certain indicators, while other studies have used various methods to create indices to measure all or some of the elements in the Bruce framework; in either case, indicators may be chosen from existing data collection tools based on the feasibility of data collection, or custom indicators may be developed anew. Where no standardized set of indicators exist, but instruments are designed to capture certain domains of FP QoC, data reduction techniques can be useful to enhance comparability and simplify analyses. For example, the authors of a 2014 DHS Analytical Study to assess the quality of care in FP, antenatal, and sick child services in several countries used principal components analysis (PCA) to create indices corresponding to structure, process, and client satisfaction ^12^. Elsewhere, factor analysis has been used to reduce indicators into variables representing different domains of quality corresponding to the Bruce framework ^13^. Exploratory Factor Analysis (EFA) is particularly useful in situations where multiple latent variables are likely to be the source of variation in a set of indicators, such as is the case in the measure of QoC. EFA can help to determine how many latent variables underlie a set of indicators, and—like PCA—provide a means for data reduction in order to explore the relationship between QoC and outcomes of interest ^14^.

Another measure used to assess FP service quality is the Method Information Index (MII). The MII aims to capture—from the client perspective—similar information to what would be measured in the Information Provision domain of the Bruce Framework. Specifically, the MII assesses FP counseling quality through three questions, asked of women in regard to the family planning visit where they received their contraceptive method: “Were you informed of potential side effects of the method?”, “Were you informed on what to do if you experienced side effects?”, and “Were you told about other methods of family planning apart from your current method?” The MII is one of 18 core indicators tracked by the Family Planning 2020 (FP2020) global partnership, which formed in 2012 following the London Summit on Family Planning to help reduce unmet need in the world’s poorest countries ^15^. It has also been used in population-based surveys, such as the Performance Monitoring and Accountability 2020 (PMA2020) surveys and the DHS Women’s questionnaire, to report on the quality of FP counseling at the national-level. In these surveys, the MII is calculated as the percentage of women who respond ‘yes’ to all three questions, whether they have received a modern contraceptive or not ^16, 17^.

The MII captures information provision during the counseling session and a woman’s understanding of having received that information. The simplicity and versatility of the MII and the fact that it can be measured in client exit interviews as well as in household surveys makes it an appealing choice for programs and clinics seeking to routinely approximate program performance. It can be aggregated and reported at multiple levels—from the clinic to national-level estimates—and it places the client perspective at the center of quality measurement. Recognition that the patient is the expert in the patient experience, or a foci of client-centeredness, has been an essential theme of three recent high level reports from U.S. National Academy of Medicine, the WHO, and the Lancet Commission on High Quality Health Systems ^18– 21^. Furthermore, it is collected and analyzed consistently where tools applying the full Bruce framework are not. However, it captures only one aspect of the Bruce framework – “information given to clients” – and, as with more comprehensive measures, there is limited evidence to show how well MII predicts outcomes of interest, such as contraceptive discontinuation, when measured at the individual level or when applied programmatically.

Recent (2014) Demographic and Health Survey data from Kenya—the site of this study—indicates that more than half (58%) of women are using contraception, up from 45.5% in 2008–09, and knowledge of modern contraceptive methods among women and men age 15 to 49 years is nearly universal; yet, 18% of currently married women have an unmet need for family planning services. Of family planning use episodes, 31% were discontinued within 12 months. Eleven percent of those episodes ended with a switch to a new method, and 11% were discontinued with no switching due to side effect and health concerns (i.e., discontinuation while in need without switching) ^22^. With regard to MII in Kenya, 60% of current users of modern contraceptive methods in 2014 reported that they were informed about potential side effects of their method, 52% were told what to do if they experienced side effects, and 79% were given information about other methods ^22^.

The present study is a secondary analysis of data collected in select urban sites of Kenya as part of the Measurement, Learning & Evaluation (MLE) Project, implemented by the Carolina Population Center at UNC Chapel Hill. The MLE Project was the evaluation component of the Urban Reproductive Health Initiative (URHI), which operated in Kenya, Nigeria, India, and Senegal from 2010 to 2015. In Kenya, the URHI project, called *Tupange*, operated in five urban areas in Kenya: Nairobi, Mombasa, Kisumu, Machakos, and Kakamega. The data used come from baseline health facility surveys and household surveys of women that were conducted in 2010 to 2011 (baseline) and again in 2014 to 2015 (endline) in these five urban areas. Two methods for assessing QoC in FP are incorporated into the surveys: the MII, and a modified version of the Quick Investigation of Quality (QIQ). The QIQ has historically used client exit interviews, facility audits, and provider observation to assess a short list of quality of care indicators at the health facility level which align with 5 domains of the Bruce Framework, mentioned previously. Facility readiness is assessed through the facility audit, while the exit interview collects information related to clients’ experience of care at the health facility ^10^. The MLE survey instruments incorporate provider surveys in place of provider observation to assess technical competence in clinical procedures and counseling skills. The MLE facility audit contains additional items to assess the sixth domain of the Bruce framework: constellation of services ^23^.

In this study, we demonstrate how existing metrics and data sources can be used to assess and improve the measurement of family planning QoC through an examination of the relationship between FP service quality and modern contraceptive discontinuation while-in-need using two established QoC measures.

## Methods

### Data sources and survey design

The MLE project was initiated in 2009 ahead of the implementation of the *Tupange* project in Nairobi, Mombasa, Kisumu, Kakamega, and Machakos. Data collection activities included a household survey and a service delivery point (SDP) assessment. The survey and sampling protocol are described below for the data included in the present analysis.

The baseline household survey was conducted with a representative sample of women in the intervention cities from September to November 2010. The household sample was drawn through a two-stage cluster sampling design in which clusters were identified from the most recent Population and Housing Census (2009) and randomly selected in each urban area, from which a random sample of 30 households per cluster was selected. Across the five intervention cities, a total of 13,140 household were selected for interviews. Women aged 15 to 49 years who were residents or household visitors were eligible to be interviewed ^24^. A follow-up household survey was conducted at endline in 2014/2015, wherein eligible women were tracked using contact information collected at baseline. Household surveys collected information on a variety of topics, including demographic information and household characteristics, current and past FP use and sources of FP. At endline, 5 year reproductive health calendars were also collected ^25^.

Baseline SDP surveys took place from August 2011 to November 2011. In Nairobi and Mombasa, all public facilities and all URHI/ *Tupange* facilities were selected; private facilities that were identified as sources of FP by women in baseline household surveys were also selected for surveys. In Kisumu, Kakamega and Machakos, all public and private facilities offering sexual and reproductive health services were included. In total, 279 facilities were surveyed. The SDP survey incorporated a health facility audit, health care provider surveys and client exit interviews. Client exit interviews were only conducted in facilities that reported offering reproductive health services routinely Women aged 15 to 49 years were approached for an interview as they exited reproductive health or child health departments. Interviewers aimed to reach 40 women per facility, half family planning clients and half clients of any other reproductive health service ^23^. Survey tools used are available as Extended data ^26^.

### Inclusion criteria

To assess service quality at the facility level, data are used only from facilities that reported offering FP services, and where provider interviews, client exit interviews, and a facility audit were conducted at baseline in 2011. 279 facilities were sampled for the baseline service delivery point assessments. Of these, 19 did not offer FP services and were excluded. Of the remaining 260 facilities, 124 had received all three assessment types and were included in the analysis

**Figure 1.**
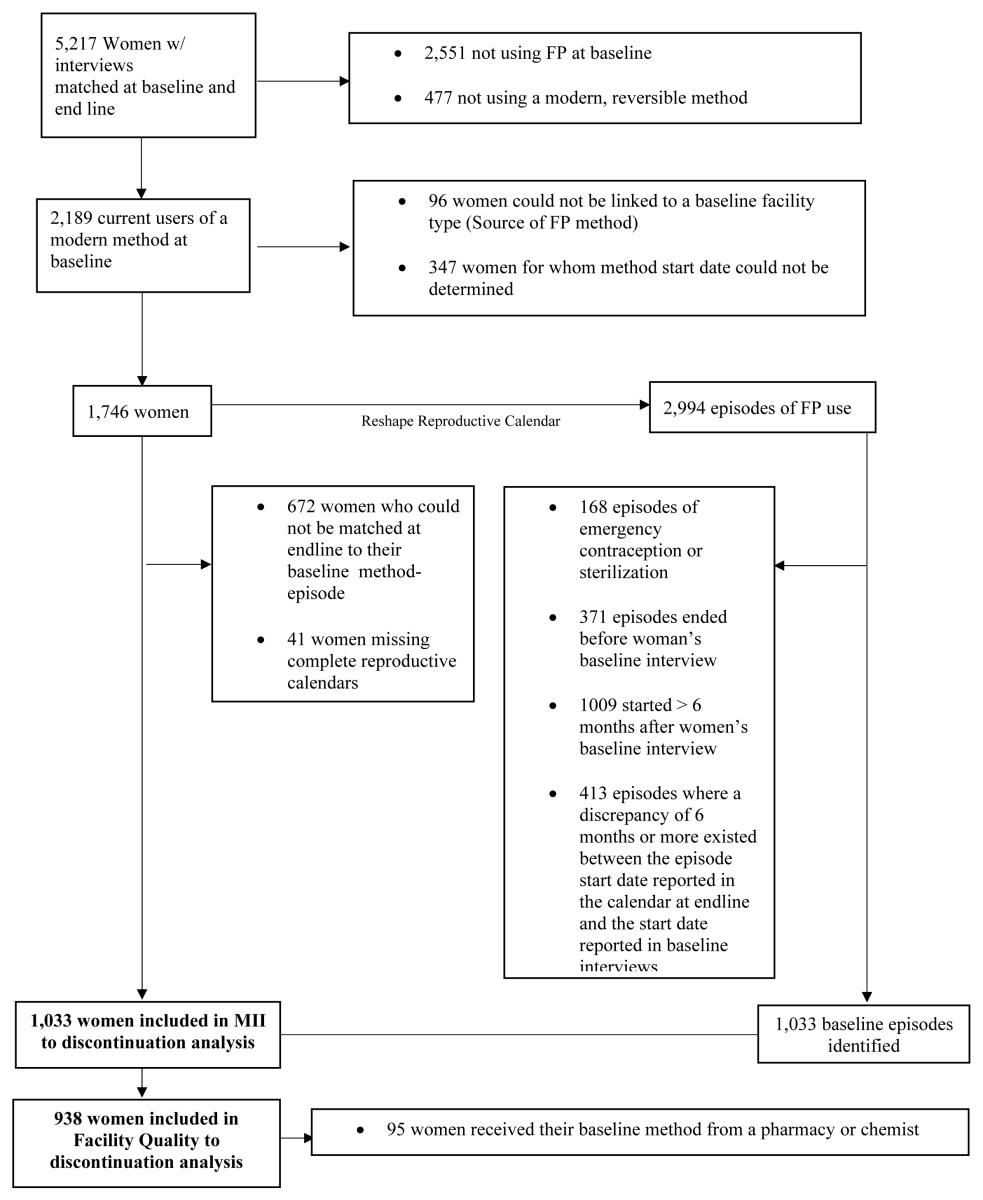
Participant Diagram.

To assess contraceptive discontinuation, individual-level data is used from matched baseline (2010/2011) and endline (2014/2015) household surveys of women. Women who were not using a modern, reversible contraceptive method at baseline were excluded, as were women for whom the method start date could not be determined and women who did not report where she received her method, or who reported that she did not know where she received her method. Women with no information on where they received their method were excluded from both models assessing the relationship between facility-quality and discontinuation and models assessing the relationship between woman-reported MII and discontinuation, because facility-type was included as a covariate in the models. For the remaining women in the sample, 5-year retrospective reproductive calendars were examined to identify the episode of contraceptive use reported by the women at baseline. Women for whom the beginning of her baseline episode could not be identified in the reproductive calendar within 6 months of the method start date reported at baseline were excluded from the analysis. Although pharmacies are recognized as part of the private health sector in Kenya, women who received their method from a pharmacy or chemist (n = 95) were excluded from models assessing the relationship between facility-level measures of quality and discontinuation but included in models assessing the relationship between woman-reported MII and discontinuation. This is because no pharmacies were included in the SDP survey; therefore, facility-level measures of quality as assessed in this study are not reflective of QoC in a pharmacy setting. Figure 1 illustrates how women were selected for inclusion in the present analyses (n = 1,033 & n = 938). Chi-square statistics were calculated to examine demographic differences between current FP users who were included and excluded in the analyses ( Table 1).

**Table 1.** Demographic characteristics and baseline contraceptive method use of women aged 15 to 49 in urban Kenya included in models assessing the relationship between the Method Information Index (MII) and discontinuation-while-in-need. Of the 2,189 users of modern, reversible contraceptive methods at baseline, 1,033 were included in the MII to discontinuation model and 1,156 current FP users were excluded. (Note: an additional 95 women were excluded from the facility quality to discontinuation model because they reported receiving their method from a pharmacy or chemist).

	Current FP users included in analysis (N = 1,033)		Current FP users excluded from analysis (N = 1,156) ^1^		
Demographic Characteristics	N	%	N	%	*p ^3^*
**Age**					
15 to 24	263	25.5%	342	29.6%	
25 to 34	535	51.8%	508	43.9%	
35 to 49	235	22.8%	306	26.5%	**<0.01**
**Education**					
No Education	13	1.3%	25	2.2%	
Incomplete Primary	155	15.0%	184	15.9%	
Complete Primary	327	31.7%	350	30.3%	
Secondary plus	538	52.1%	597	51.6%	0.36
**Wealth ^2^**					
Quintile 1	255	24.7%	275	23.8%	
Quintile 2	221	21.4%	254	22.0%	
Quintile 3	184	17.8%	229	19.8%	
Quintile 4	222	21.5%	251	21.7%	
Quintile 5	151	14.6%	147	12.7%	0.58
**City**					
Nairobi	256	24.8%	342	29.6%	
Mombasa	109	10.6%	168	14.5%	
Kisumu	154	14.9%	235	20.3%	
Machakos	313	30.3%	227	19.6%	
Kakamega	201	19.5%	184	15.9%	**<0.01**
**Marital Status**					
Never married	60	5.8%	187	16.2%	
Married or living together	902	87.3%	856	74.1%	
Other	71	6.9%	113	9.8%	**<0.01**
**Parity**					
No births	7	0.7%	47	4.1%	
One birth	135	13.1%	174	15.1%	
Two births	358	34.7%	306	26.5%	
Three births	268	25.9%	291	25.2%	
Four or more births	265	25.7%	338	29.2%	**<0.01**

^1^ Current users of family planning (FP) who were excluded from analysis if they matched any of the following criteria: 1) start date of baseline method could not be determined; 2) woman could not be linked to facility type as the source of her method at baseline; 3) complete reproductive calendar information was not available for the woman at end line; or 4) the woman’s baseline episode of contraceptive use could not be identified in her reproductive calendar within 6 months of the start date she reported at baseline.

^2^ Wealth quintiles are calculated across the 5 cities.

^3^ A chi-square test of independence was performed to examine differences between current FP users included or excluded from the analysis.

### Statistical analysis

All analyses were conducted using Stata version 14.2 ^27^.

### Linking women to facility level measures of quality

To examine the relationship between facility-level measures of quality and discontinuation, facilities were categorized as public hospitals, public facilities, private hospitals and private facilities. Public facilities included government health centers, government dispensaries and other public facilities. Private facilities included private clinics, nursing/maternity homes, faith-based home/health centers, other NGO clinics, and other private facilities. In baseline household interviews, women reported the facility type where they received their method, and those facility types were condensed into 4 categories to align with the SDP analysis. Because women could not be linked to the specific facility where they received their baseline contraceptive method, quality variables were aggregated into categories according to city and facility type, which necessitates the assumption that women would have attended a facility within the city where they lived. Where facility audits distinguished between private hospitals and private clinics, the household survey combined private and faith-based hospitals and clinics into two categories: ‘Private hospital/clinic’ and ‘Faith-based hospital/clinic’. Women who reported receiving their method from one of these categories, were linked to measures of quality at private hospitals, except in Kakamega – where no private hospitals were included in the SDP assessment, so women were linked to measures of quality at private facilities.

### Discontinuation analyses

Three models were constructed to determine the relationship between measures of FP service quality and discontinuation: two to assess MII reported at baseline in household interviews (as an ordinal variable, and as a binary variable), and one for summative domains of quality at the facility level. Women who indicated that they discontinued their method due to side effects, health concerns, method failure, issues related to access or disapproval of their partner and who did not switch to a new modern method were considered to have discontinued while in need. Women who indicated that they wanted to become pregnant, were no longer sexually active, or who switched to a different modern method were censored. Time to discontinuation was measured in months. All women were right-censored at 36 months. For all discontinuation models, Cox proportional hazards ratios were estimated where the event of interest was discontinuation while in need without switching. Individual and facility type variables were screened for inclusion as covariates in the final adjusted models. Individual characteristics at baseline – age, marital status, parity, education, wealth and method-type (long-term method or short term method— were considered as potential confounders of the relationship between quality and contraceptive use. These characteristics may directly impact both a woman’s likelihood of continued contraceptive use and the way she is treated by providers and staff at a facility. In models to assess the relationship between MII and discontinuation, pharmacies were categorized as ‘Private Facilities’ within the facility type variable. Correlations between potential covariates were examined. All independent variables and covariates were checked to ensure proportional hazards assumptions were met. The final models of women-level and facility-level measures of quality related to discontinuation were adjusted for covariates that were significant at p < 0.1 when examined individually in a Cox proportional hazard model. We accounted for intragroup correlation in the facility-level discontinuation analysis by using a shared frailty model ^28^. Facility level quality domains were assessed first individually, and then together.

### Independent variables

***Calculation of Woman MII.*** Women were assigned an MII score of 0 to 3 by adding together her responses to each of the three MII questions (0 for no, 1 for yes), asked during baseline household interviews in reference to her current method: “Were you told by a health or family planning worker about side effects or problems you might have using this family planning method?”, “Were you told by a health or family planning worker about what to do if you experienced side effects or problems with this method?”, “Were you told by a health or family planning worker about other methods of family planning (beside the one you are currently using)?” Woman MII was examined in discontinuation analyses as an ordinal variable (0 to 3) and as a binary variable (3 vs. less than 3).

***Facility quality variables.*** Exploratory factor analysis (EFA) was used to identify domains of family planning service quality at the facility level. To incorporate data from provider interviews, a single representative provider survey was chosen for each facility based on highest cadre, and most years worked at the facility, rather than choosing to average the values across providers within the facility. The number of provider surveys collected at each facility was not consistent or representative, so these criteria were used to incorporate the responses of those providers who were most likely to be knowledgeable of overall facility operations. Client exit interview variables were incorporated into the EFA as continuous variables (0 to 1) corresponding to the proportion of clients at the facility who indicated an affirmative response to each question. Efforts were made to reduce the number of variables entered into the EFA model, in order to improve the subject to item ratio (N:p) ^29^. Variables were combined into composite variables aligned with the list of 25 QIQ indicators where feasible, and converted to binary variables or standardized to continuous variables (0 to 1) ^10^. Variables pertaining to basic infrastructure – water, electricity and toilet facilities – were combined into a binary variable and assigned a value of 1 if the facility had all three characteristics. To generate a standardized method-mix score, facilities received one point each for having available: one permanent, one long-acting reversible contraception (LARC) (implant or intrauterine device (IUD)), one short term hormonal method (pill, emergency contraception (EC), injectable), and one barrier method. The number of family planning methods offered was summed (range: 0 to 12) for each facility, and then standardized to 0 to 1. Checklist items not included in the list of 25 QIQ indicators, such as those pertaining to infection prevention equipment, were standardized to a continuous variable (0 to 1) according to the proportion of items achieved within the checklist. Other facility audit variables were coded as binary variables. The final N:p ratio was 124: 38, or 3.3:1.

The EFA was an iterative process. All variables were entered into the factor analysis model using the principal factors method. The number of factors was determined based on the resulting scree plot, and by restricting the analysis to factors with eigenvalues > 1 ^29^. Because the domains of FP QoC are theoretically related, oblique factor rotation was applied in order to allow for correlations between factors. Factor analysis was repeated until all variables loading with a uniqueness > 0.8 were excluded, and those remaining loaded on to at least one factor at 0.3 or higher. Variables were most often assigned to the factor where they loaded the highest. In instances where variables loaded highly onto more than one factor, assignment decisions were informed by the other contents of each factor. Cronbach’s alpha was examined for each factor to understand the internal consistency of the items contained within ^14^. Summative quality domain variables were generated for each facility by adding up the values of the variables that loaded into each factor.

### Facility level quality to MII analysis

To better understand the relationship between these two measures of FP service quality that could be collected at the facility level, a facility-level measure of MII was calculated for each of the 124 facilities included in the quality analysis. Facility MII was calculated at the percent (%) of FP clients who responded ‘yes’ to all three MII questions in exit interviews conducted during the baseline SDP survey. Spearman correlations were run between facility-level MII and each summative quality domain among the sample of facilities included in the analysis. Additionally, a scatter plot fitted with a regression line and 95% CI was examined for facility-level MII vs. overall quality scores.

## Results

### Analytical sample

One hundred and twenty four (124) facilities were included in the present analysis. Table 2 describes the distribution and characteristics of facilities included in the analysis. Approximately 53% were public hospitals or other public facilities; 47% were private hospitals or other private facilities. The majority of facilities—approximately 35%—were located in Nairobi. Of 5,217 women with matched baseline and endline interviews, 1,033 women were ultimately included in the MII to discontinuation analysis and 938 were included in the facility quality to discontinuation analysis. The resulting analytical samples consisted of 1,033 and 938 baseline episodes of contraceptive use (one per woman), respectively. Table 1 describes the demographic characteristics of current FP users (n = 2,189) who were included in (n = 1,033) and excluded from (n=1,156) the MII to discontinuation analysis. There were no significant differences in age (p = 0.82), education (p = 0.49), wealth (p = 0.45), or parity (p = 0.59) between current FP users who were included and excluded from the analyses; however, current FP users who were excluded from the analysis were more likely to have never married (p < 0.05). The salient features of the episode of contraceptive method use at baseline among women included in the analyses can be found in Table 3. Most women received their method from a public hospital (44.9% and 49.5%, respectively) or private hospital (26.2%, and 25.7%, respectively). The majority of women used injectables (59.2% and 62.5%, respectively) or oral contraceptives (19.0% and 14.7%, respectively) as their contraceptive method. 20.9% and 21.1% of baseline FP users in each of the two analytical samples discontinued use of their method within 3 years; 5.2% and 4.9% switched to a different method; 73.9% and 74.0% continued use of their method throughout the 3 year window.

**Table 2.** Distribution and facility type of 124 high-volume facilities included in the quality analysis.

City	Nairobi		Mombasa		Kisumu		Machakos		Kakamega		Total	
Facility Type	n	%	n	%	n	%	n	%	n	%	n	%
Public Hospital	4	3.2	3	2.4	2	1.6	1	0.8	1	0.8	11	8.9
Other Public Facility	27	21.8	11	8.9	12	9.7	2	1.6	3	2.4	55	44.4
Private Hospital	1	0.8	2	1.6	4	3.2	1	0.8	0	0.0	8	6.5
Other Private Facility	11	8.9	9	7.3	12	9.7	11	8.9	7	5.7	50	40.3
**Total**	43	34.68	25	20.15	30	24.2	15	12.1	11	8.9	124	100.0

**Table 3.** Characteristics of baseline contraceptive use for current FP users included in models assessing the relationship between method discontinuation and the Method Information Index (MII) or Facility Quality, respectively.

	MII to Discontinuation Models (N = 1,033)		Facility Quality to Discontinuation Models (N = 938)	
Baseline Contraceptive Use	n	%	n	%
**Method**				
Implant	120	11.6%	119	12.7%
IUD	73	7.1%	70	7.5%
Injectable	611	59.2%	586	62.5%
Oral Contraceptive	196	19.0%	138	14.7%
Condoms	33	3.2%	25	2.7%
**Episode Type (3YR) ^1^**				
Discontinuer	216	20.9%	198	21.1%
Switcher	54	5.2%	46	4.9%
Continuer	763	73.9%	684	74.0%
**Source of FP Method**				
Public Hospital	464	44.9%	464	49.5%
Other Public Facility	191	18.5%	191	20.4%
Private Hospital	241	23.3%	241	25.7%
Other Private Facility	137	13.7%	42	4.5%
**MII Score**				
0	162	15.7%	140	14.9%
1	217	21.0%	184	19.6%
2	81	7.9%	77	8.2%
3	573	55.5%	537	57.3%
**MII by Question**				
Informed of side effects	669	64.8%	627	66.9%
Told how to resolve problems	611	59.2%	572	62.0%
Informed of other methods	818	79.2%	750	80.0%

^1^Classification of the baseline episode at 3 years. Discontinuers are women who discontinued their method within 3 years for any reason, without switching to a new method – including discontinuation while in need, discontinuation while not in need, and method failure. IUD – intrauterine device, FP – Family planning

**Figure 2.**
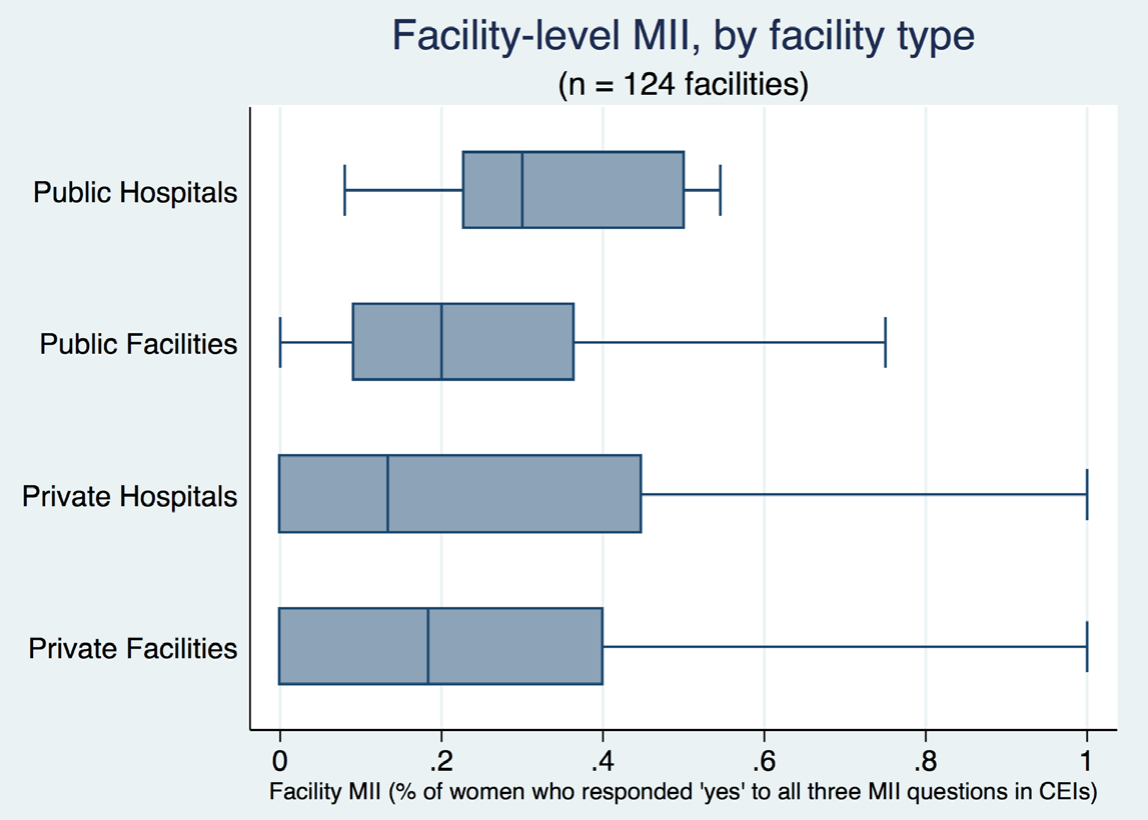
Distribution of facility-level Method Information Index (MII) scores, by facility type. Minimum, median and maximum scores are indicated.

The distribution of women’s MII scores is presented at the bottom of Table 3. In baseline household interviews, 15.7% of the 1,033 women included in the MII to discontinuation analysis reported never having been informed of any the three elements of the MII in regard to their current method. More than half of the sample (55.5%) answered ‘Yes’ to all three questions. There were no significant differences in the distribution of MII scores (p = 0.26) or the frequency with which women answered yes to all three MII questions between the full analytical sample and the reduced sample used in the facility quality to discontinuation analysis (p = 0.33). Figure 2 presents overall facility-level MII, by facility type, for the 124 facilities included in analysis.

### Quality analysis

The following six domains of quality were identified in EFA of 38 facility-level variables within the sample of 124 facilities: client satisfaction, readiness for choice and management support; infrastructure & equipment; privacy & comfort; information exchange; and technical competence ( Table 4). The total number of variables was reduced from 38 to 25. The actual range and distribution of summative facility quality domain scores across the 124 facilities included in the analysis are described in Table 5.

**Table 4.** Results of exploratory factor analysis (EFA) of facility-level quality variables from 124 high-volume facilities.

Domain (α)	Variable	Data Source	Factor Loading
**Client Satisfaction (0.79)**	Proportion of clients who felt they were treated very well by the providers at the facility	Client Exit Interview	0.9610
	Proportion of clients who felt they were treated very well by other staff at facility	Client Exit Interview	0.6917
	Proportion of clients for whom the provider demonstrated good counseling skills	Client Exit Interview	0.8179
**Readiness for Choice &** **Management Support (0.65)**	Total # FP Methods Offered	Facility Audit	0.7912
	Standardized Method Mix Available Score	Facility Audit	0.4956
	Written guidelines/protocols for FP services in place	Facility Audit	0.6489
	Written guidelines/protocols for integration of FP and HIV services in place	Facility Audit	0.4531
	Written guidelines or tools to screen patients for pregnancy in place	Facility Audit	0.3289
	Audits or reports are conducted at least quarterly	Facility Audit	0.3363
**Information Provision (0.89)**	Proportion of clients who felt the information they received during their visit was at least enough	Client Exit Interview	0.4020
	Proportion of clients who discussed side effects with provider at facility	Client Exit Interview	0.6014
	Proportion of clients who discussed what to do when they have problems with the provider at the facility	Client Exit Interview	0.5930
	Proportion of clients asked about reproductive goal at the facility	Client Exit Interview	0.4749
**Infrastructure & Equipment** **(0.63)**	Facility has all equipment and supplies necessary to deliver methods offered	Facility Audit	0.6454
	Private examination room	Facility Audit	0.4675
	Facility infection prevention equipment (out of those surveyed)	Facility Audit	0.6040
	Facility has basic infrastructure (electricity, running water, toilet facilities)	Facility Audit	0.3810
**Privacy & Comfort (0.53**)	Proportion of clients who felt that they had visual privacy	Client Exit Interview	0.6407
	Proportion of clients who felt that they had audio privacy	Client Exit Interview	0.6197
	Proportion of clients who felt comfortable asking questions	Client Exit Interview	0.4760
**Technical Competence** **(0.50)**	Provider keeps personal/financial records - a client record card/form	Provider Survey	0.3659
	Provider helps client select a suitable method	Provider Survey	0.3452
	Provider explains the way to use the selected method	Provider Survey	0.5443
	Provider explains the side effects of the selected method to clients	Provider Survey	0.3945
	Provider discusses the client's family planning preferences	Provider Survey	0.4299

**Table 5.** Range and distribution of summative facility quality domain scores, summarized by city and by facility type.

Category	n	Client Satisfaction (range: 0.0 to 3.0)			Readiness for Choice & Management Support (range: 0.0 to 6.0)			Infrastructure & Equipment (range: 0.0 to 4.0)			Privacy & Comfort (range: 0.0 to 4.0)			Information Provision (range 0.0 to 4.0)			Technical Competence (range 0.0 to 5.0)		
		mean	sd	range	mean	sd	range	mean	sd	range	mean	sd	range	mean	sd	range	mean	sd	range
**All**	124	0.9	0.7	0.0 – 3.0	3.9	1.4	0.5 – 6.0	2.9	1.0	0.5 – 4.0	2.7	0.7	0.5 – 4.0	2.3	0.7	0.0 – 4.0	3.2	1.3	0.0 – 5.0
**City**																			
Nairobi	43	0.8	0.6	0.0 – 2.3	4.4	1.1	2.1 – 6.0	3.1	0.9	1.2 – 4.0	2.9	0.5	1.9 – 3.8	2.5	0.6	1.5 – 3.8	3.2	1.3	0.0 – 5.0
Mombasa	25	1.1	0.7	0.0 – 2.5	4.3	1.2	1.7 –5.9	2.7	1.0	0.7 – 4.0	2.4	0.5	1.7 – 3.5	2.0	0.6	1.0 – 3.0	2.7	1.2	1.0 – 5.0
Kisumu	30	0.6	0.8	0.0 – 2.5	3.5	1.5	0.6 – 5.8	2.7	1.2	0.5 – 4.0	2.6	0.9	0.5 – 4.0	2.1	0.9	0.0 – 4.0	3.4	1.5	0.0 – 5.0
Machakos	15	1.3	0.8	0.0 – 3.0	2.7	1.6	0.5 – 5.8	3.1	0.8	2.0 – 4.0	2.6	0.7	1.5 – 4.0	2.1	0.8	1.0 – 4.0	3.4	1.0	2.0 – 5.0
Kakamega	11	1.4	0.9	0.1 – 3.0	3.9	1.2	2.2 – 5.8	3.2	1.1	1.2 – 4.0	2.9	0.8	1.7 – 4.0	2.6	0.7	1.4 – 4.0	3.2	0.6	2.0 –4.0
**Facility Type**																			
Public Hospitals	11	0.8	0.4	0.2 – 1.4	5.2	0.8	3.4 – 6.0	3.7	0.6	2.7 – 4.0	2.9	0.5	1.9 – 3.3	2.4	0.5	1.3 – 2.9	3.7	1.5	0.0 – 5.0
Public Facilities	55	0.8	0.6	0.0 – 2.5	3.9	1.2	0.6 – 5.6	2.5	1.0	0.5 – 4.0	2.5	0.6	0.5 – 3.8	2.2	0.6	0.5 – 3.3	3.1	1.4	0.0 – 5.0
Private Hospitals	8	0.7	0.8	0.0 – 1.7	4.5	1.0	2.1 – 5.5	3.7	0.6	2.8 – 4.0	2.9	0.8	2.0 – 4.0	2.4	0.9	1.0 – 4.0	2.8	1.3	1.0 – 5.0
Private Facilities	50	1.1	0.9	0.0 – 3.0	3.5	1.5	0.5 – 6.0	3.1	1.0	0.8 – 4.0	2.9	0.8	0.5 – 4.0	2.4	0.9	0.0 – 4.0	3.2	1.1	0.0 – 5.0

Note: Summative quality domain are summarized here for descriptive purposes by city and by facility type; however, scores were applied to women in discontinuation analysis according to city-facility type categories (i.e. public hospitals in Nairobi).

### Facility level MII to facility level quality

At the facility-level, MII measured as the % of women who responded ‘yes’ to all three MII questions in client exit interviews was significantly correlated with the domains of infrastructure & equipment (R = 0.235, p < 0.05), privacy & comfort (R = 0.682, p < 0.001, and information provision (R = 0.676, p < 0.001) ( Table 6).

**Table 6.** Correlation between facility-level Method Information Index (MII) scores and domains of family planning quality derived in exploratory factor analysis (EFA) (n = 124 facilities).

Domain	Spearman’s rho	p
Client Satisfaction	0.119	0.190
Readiness for Choice & Management Support	0.117	0.196
Infrastructure & Equipment	0.235	**0.009**
Privacy & Comfort	0.682	**< 0.001**
Information Provision	00.676	**< 0.001**
Technical Competence	-0.001	0.989

### Discontinuation analysis

Among the 216 women who stopped using their method within the 3-year period, 210 discontinued while-in-need (20.3% of 1,033 women included in the MII to discontinuation analysis). The results of the discontinuation analysis are presented in the form of crude and adjusted hazard ratios in Table 7 and Table 8. All models are adjusted for age, method-type and facility-type.

**Table 7.** Unadjusted and adjusted hazard ratios for 3 -year discontinuation of modern contraception while-in-need, by Method Information Index (MII) score reported by woman at baseline; two models presented for binary and ordinal variables (n = 1,033 women).

Variable	Unadjusted			Adjusted ^1^		
	HR	p	[95%Conf. Interval]	HR	p	[95 % Conf. Interval]
**Method Information Index (Binary)**						
Answered ‘No’ to at least one question (ref)						
Answered ‘Yes’ to all 3 questions	0.76	0.180	[0.50 1.14]	0.79	0.257	[0.52 1.19]
**Method Information Index (Ordinal)**						
MII – 0 (Answered ‘No’ to all 3) (ref)						
MII - 1	0.45	0.012	[0.24 0.84]	0.45	**0.014**	[0.24 0.85]
MII - 2	0.45	0.075	[0.19 1.09]	0.51	0.142	[0.21 1.25]
MII - 3	0.48	0.003	[0.23 0.78]	0.51	**0.009**	[0.31 0.84]

^1^Adjusted for woman’s age at baseline, facility type, and method type (long-term vs. short-term method)

**Table 8.** Unadjusted and adjusted hazard ratios for 3-year discontinuation-while-in-need of modern contraception by facility-level measures of quality (n = 938 women).

Variable	Unadjusted			Adjusted		
	HR	p	[95%Conf. Interval]	HR	p	[95 % Conf. Interval]
Privacy & Comfort	0.41	0.255	[0.09 - 1.89]	0.62	**<0.001**	[0.48 - 0.81]
Technical Competence	0.7	0.973	[0.73 - 1.35]	0.7	**0.037**	[0.50 - 0.98]
Client Satisfaction	1.76	0.074	[0.95 - 3.28]	3.36	**0.006**	[1.42 - 7.96]
Information Provision	1.05	0.874	[0.56 - 1.99]	0.66	**0.016**	[0.46 - 0.93]
Infrastructure & Equipment	0.65	0.080	[0.4 - 1.05]	0.63	0.281	[0.27 - 1.46]
Readiness for Choice & Management Support	1.2	0.111	[0.96 - 1.50]	1.07	0.583	[0.84 - 1.37]

^1^Intragroup correlation was accounted for using a shared frailty model

^2^Adjusted for woman’s age at baseline, facility type, and method type (long term vs. short term method)

Women who responded yes to all three MII questions asked in relation to their current contraceptive method in baseline household interviews were no less likely to discontinue their method while in need than women who responded yes to less than three (p > 0.05); however, when MII was examined as an ordinal variable in the discontinuation analysis, a woman’s likelihood of discontinuation while in need was reduced by approximately 50% whether she reported being informed of just one aspect of MII (HR: 0.45, p < 0.05), or informed of all three (HR: 0.51, p < 0.01) compared to none ( Table 7) versus those who received no information. Facility-level measures of quality derived through EFA were found to be significantly associated with discontinuation as well ( Table 8). Higher scores in the domains of privacy & comfort (p < 0.001), technical competence (p < 0.05), and information exchange (p <0.05) reduced the risk of discontinuation while in need without switching; higher scores in client satisfaction were associated with an increased risk of discontinuation (p < 0.01). A stratified analysis was conducted to examine the possibility of effect modification by provider-controlled versus woman-controlled methods on the relationship between client satisfaction and discontinuation while in need (Extended Data). The finding remains significant among women who are not using provider controlled methods but is no longer significant among women who are using provider controlled methods.

## Discussion

In this study of women and facilities in urban Kenya, two measures of FP service quality were developed and assessed for their association with contraceptive discontinuation while in need without switching: constructed indicators from a QIQ-based facility quality assessment tool and the Method Information Index (MII). Women who reported receiving higher quality FP counseling according to their MII score were significantly less likely to discontinue their method over the next three years compared to women with an MII score of zero. In fact, a woman’s likelihood of discontinuation while in need without switching to a new method was cut in half whether she reported being informed of just one aspect of MII or informed of all three versus receiving no information at all.

The analysis of the comprehensive facility-based QoC assessment tool was more complex. Using EFA, we identified six domains of FP service quality captured by the assessment tool. Four of these domains – client satisfaction, readiness for choice & management support, information exchange, and technical competence—closely align with the domains that the QIQ indicators are intended to capture; however, two additional domains of quality emerged in this setting: privacy & comfort, and infrastructure & equipment. Items included in the assessment tool which may have captured mechanisms for follow-up and/or constellation of services were not sufficiently correlated to be identified as domains of quality in this analysis. Of the six that were identified, three domains of quality—privacy & comfort, technical competence, and information exchange—were found to be significantly associated with a decreased risk of contraceptive discontinuation.

Here, we also demonstrate how existing metrics and data can be used to assess and improve the measurement of quality of care related to family planning health outcomes, as others have done with MLE data from Kenya. Prior studies in Kenya and elsewhere have described the quality of family planning service delivery and have assessed the relationship between quality of care and current contraceptive use, but to our knowledge, few have assessed quality of care against contraceptive discontinuation while-in-need as an outcome of interest ^13, 30– 32^. Furthermore, while many studies have examined aspects of client-centered counseling and how they relate to contraceptive discontinuation across a variety of contexts, few studies look specifically at the MII. In a prospective study of women in Pakistan and Uganda, Chakraborty *et al*. similarly found that baseline MII scores were positively associated with method continuation rates among clients in social franchises ^33^. By leveraging this existing dataset, which includes clients at both private and public sector facilities, we add depth to a growing body of work on the potential use of the MII as an indicator of contraceptive counseling quality.

Programs operating in urban Kenya that wish to reduce the rate of contraceptive discontinuation while in need among their clients might refer to these preliminary findings in their decision making. When women receive, or understand to have received, the information reported in the MII, they are less likely to discontinue their method without switching to another method. Thus, programs may choose to prioritize ensuring provision of full information on other methods and side effects in all contraceptive counseling that takes place at a facility and from this, could expect some reduction in each patient’s risk of discontinuing her method as a result. Additionally, efforts to improve aspects of privacy & comfort in FP service provision and to hire and train competent providers may strengthen rates of contraceptive continuation among clients. These results echo findings from other settings where current method use or discontinuation were the outcome of interest. For example, interventions to train providers in evidenced-based medicine or to improve provider competencies in areas such as contraceptive counseling and client-management have been shown to improve contraceptive continuation in a variety of settings ^34– 36^. Similarly, a 2002 study conducted in Egypt found that interactions between providers and family planning clients that could be characterized as ‘client-centered’ versus ‘physician-centered’ were associated with higher rates of method continuation 7 months later ^37^.

As such, these findings may contribute to efforts to develop standardized, actionable measures for QoC in FP. The framework established by Bruce in 1990 ushered in a client focused approach to family planning services by establishing quality services as those in which clients are free to choose a method, are empowered to continue using the method, and are provided with avenues to seek help with or change their method. The framework achieved this paradigm shift by emphasizing choice, information provision, and mechanisms for follow-up as key tenets of quality, and establishing interpersonal relationships—vital for the facilitation of those tenets of quality—as a domain of quality in itself. However, the Bruce framework does not explicitly address issues of privacy and comfort, nor are these issues explicitly addressed in many of the existing studies linking client-centered or person-centered approaches to care to FP outcomes ^37– 39^. For example, in the 2002 study in Egypt referenced above, ‘client-centered’ models of communication were identified as those with a high proportion of solidarity statements (versus disagreement statements), but issues of dignity, respect or privacy were not explicitly measured. In the present study, the domain most closely aligned with client-centered approaches to care contains explicit indicators of client comfort and privacy, including: ‘proportion of clients who reported they were comfortable asking questions’, and ‘proportion of clients who had visual privacy’, ‘proportion of clients who had auditory privacy’. The QIQ was based on the Bruce framework, and does not contain all of the aspects now considered important to client-centered care; yet, those measures that are present came together in EFA to form a domain independent of other domains of quality, even if it does not represent a comprehensive measure of client-centered or person-centered care. Given these findings, and an understanding of the rights of patients to dignified and respectful FP care, we agree with others who have suggested that more comprehensive and explicit measures of client-centeredness should be developed and incorporated into family planning quality measures ^40, 41^.

In this study, structural aspects of quality, such as infrastructure, equipment and facility readiness, were not associated with contraceptive discontinuation. Overall, these domains were more homogenous across facilities, which may suggest that indicators pertaining to structural quality, while generally easier and less expensive to measure, are better suited for measuring against a minimum acceptable level of quality, and process measures are more appropriate for teasing out which aspects of a facility or program’s performance will be correlated with better outcomes.

The appeal of using the MII over more comprehensive measures of FP QoC for routine quality monitoring at the facility or program level is clear – it contains only three questions, it is easily assessed and analyzed, it can be aggregated for upward reporting and measurement, and it allows for benchmarking against national standards. Therefore, to better understand the utility of using MII at the facility-level, we examined the correlation between facility-level measures of MII and other facility-level measures of quality. MII assessed at the facility level was not correlated with all of the domains of quality that were associated with a decreased risk of discontinuation among women. Expectedly, facility-level MII was found to be correlated with the information exchange domain. Additionally, it was correlated with scores in the facility infrastructure domain and in the privacy & comfort domain. The client exit interviews (CEI) included in the SDP assessment in the MLE study are a good fit in the context of this comprehensive assessment tool, but were not designed to be representative of FP clients at any given facility, which informed our decision to exclude facility-level MII from the discontinuation analyses. Nonetheless, given these findings – that for a woman, her understanding of having received any MII information is associated with her being less likely to discontinue her method, and that when measured at the facility level, MII is correlated with several other domains of facility quality, more research is needed to determine how the measurement of MII can be refined at the facility level so that it can provide information to facilities and program managers that is actionable for improving outcomes.

Limitations of this study include the size of the analytical sample used (n = 1,033), which represents less than half of the 2,189 women interviewed at baseline and endline who were identified as current users of a modern reversible contraceptive method in 2010. The women who were dropped would have been eligible for inclusion in the analysis had it been possible to link them to the type of facility where they had received their method, to determine their method start date, and to identify their baseline episode of contraceptive use within the reproductive health calendar collected at endline. The final samples are much smaller than the original representative sample of women, and the generalizability of these results may be limited; however, the demographic characteristics of current FP users included in and excluded from the analysis were found to be comparable ( Table 1). We were unable to identify the exact facility where each woman received her FP method; thus, this study linked women to a measure of average facility quality based on their location and the type of facility where they received her method, assuming that women seek services within the city where they reside, which may be inaccurate in some cases. Additionally, quality measures aggregated at the facility type and city level may not be representative of a woman’s individual experience of care.

Several of the factors identified in EFA have Cronbach’s alpha values below 0.7, suggesting weak scales; however, refinement of these items into definitive sub-scales and scales was not the main goal of the paper. Finally, for some women, data on facility quality were collected up to a year after baseline data on current contraceptive use were collected. We chose not to exclude women whose reported baseline episodes may have begun before SDP assessments occurred, because we do not expect that quality at the SDP changed much during that time. Together these limitations may attenuate any existing associations between other domains of facility quality identified in our study and contraceptive discontinuation.

The reason for the association identified between the client satisfaction domain and an increased risk of discontinuation is unclear, but we do not interpret this to mean that that higher rates of client satisfaction will lead to higher rates of discontinuation among women. There was no single variable within the domain that could be identified as the main driver of this finding. We considered the possibility that some women may continue contraception, particularly long-acting methods, due to coercion or pressure from providers. A stratified analysis to examine the possibility of effect modification by provider-controlled versus woman-controlled methods did not explain the finding (Extended Data). The domain includes only client reported measures (of wait time, being treated well by staff and providers, and of experiencing good counseling skills during the visit). Although our factor analysis process grouped these items together, other frameworks include these measures in different conceptual areas. For example, Jain *et al*. would not consider wait time an indication of process quality, while Hutchinson *et al*. defined client satisfaction very broadly, encompassing aspects of our domains of client satisfaction, privacy and comfort, and information provision ^40, 42^. Given that other studies have found conceptual and mathematical relationships between client reported measures and observed quality, this particular finding is not well explained within the present study.

## Conclusion

Our study found a positive association between woman-reported MII and facility-level measures of information provision, technical competence, privacy, autonomy & dignity and contraceptive method continuation over 3 years. The findings suggest that family planning facilities and programs should emphasize information provision and client-centered approaches to care alongside technical competence in the provision of FP care. More work is needed to determine how the measurement of MII can be refined at the facility level as an actionable metric for improving outcomes. Furthermore, comprehensive and explicit measures of client-centeredness which incorporate aspects of privacy, autonomy & dignity should be emphasized and operationalized as standard measures of FP QoC are advanced.

## Ethical considerations and consent

The study protocol and tools were approved by the Institutional Review Board at the University of North Carolina at Chapel Hill and the Kenya Medical Research Institute Ethical Review Committee. All household-level participants and client exit interview participants provided verbal consent while providers provided written consent.

## Data availability

### Underlying data

The data used in this study are available upon request. Please see: Carolina Population Center Data Portal for the Measurement, Learning & Evaluation

project at: https://data.cpc.unc.edu/projects/14/view to request the data.

Data are available for download after an approval of a restricted use application, which involves signing a data use agreement and providing brief information about intent of use (investigator information, research team information, and statement of purpose). The endline Women’s data is restricted to matched women only.

This project contains the following underlying data:

Baseline Service Delivery Point Survey (2010–11)

- SDP Facility Audit
- SDP Client exit interviews
- SDP Provider survey

Baseline Household Survey (2010–11)

- Women’s data

Endline Household Survey (2014–15)

- Women’s data

### Extended data

Harvard Dataverse: MLE Kenya Survey Tools. https://doi.org/10.15139/S3/XAJYU1

This project contains the following Extended data:

- Kenya Baseline Woman Questionnaire.pdf (Baseline household woman survey available in English and Swahili)
- Kenya Baseline SDP Exit Interview (Eng-Swa).pdf (Exit interview survey available in English, Swahili, Kamba and Luo)
- Kenya Baseline SDP Facility Audit.pdf (Baseline service delivery point survey)
- Kenya Baseline SDP Service Provider Survey.pdf (Provider survey available in English)

Kenya Endline Woman Quesionnaire.pdf (Endline household woman survey available in English and Swahili) Open Science Framework:

- Measures of family planning service quality associate with contraceptive discontinuation: an analysis of Measurement, Learning & Evaluation (MLE) project data from urban Kenya. The supplemental analysis to examine the relationship between the Client Satisfaction domain and discontinuation among clients using provider-controlled versus not provider-controlled methods is available here: DOI https://doi.org/10.17605/OSF.IO/BDN5E.

Data are available under the terms of the Creative Commons Zero "No rights reserved" data waiver (CC0 1.0 Public domain dedication).
