# Supplementary material for: Measures of family planning service quality associated with contraceptive discontinuation: an analysis of Measurement, Learning & Evaluation (MLE) project data from urban Kenya
Source: Gates Open Res. 2020 Jan 29;3:1453. Originally published 2019 May 2. [Version 2] doi: 10.12688/gatesopenres.12974.2 (PMC7042708; doi:10.12688/gatesopenres.12974.2)
Supplement: Peer review history

# Peer review: Reviewer response for version 2

**DOI:** 10.21956/gatesopenres.14267.r28517

**Published:** 2020-02-25

**Authors:** Kelsey Holt

**Affiliations:** 1 Department of Family and Community Medicine, University of California, San Francisco, San Francisco, CA, USA

I have reviewed the authors’ responses and I’m satisfied with their revisions.

I confirm that I have read this submission and believe that I have an appropriate level of expertise to confirm that it is of an acceptable scientific standard.

# Peer review: Reviewer response for version 2

**DOI:** 10.21956/gatesopenres.14267.r28516

**Published:** 2020-02-20

**Authors:** Amy O. Tsui

**Affiliations:** 1 Department of Population, Family and Reproductive Health, Johns Hopkins Bloomberg School of Public Health, Baltimore, MD, USA

I have read the revised version and the authors’ responses to my review. The revision is acceptable for indexing, with no additional comments.

I confirm that I have read this submission and believe that I have an appropriate level of expertise to confirm that it is of an acceptable scientific standard.

# Peer review: Reviewer response for version 1

**DOI:** 10.21956/gatesopenres.14081.r27118

**Published:** 2019-06-11

**Authors:** Amy O. Tsui

**Affiliations:** 1 Department of Population, Family and Reproductive Health, Johns Hopkins Bloomberg School of Public Health, Baltimore, MD, USA

The Method Information Index is one of the core FP2020 indicators tracked through surveys based on client experience with provider, in being told of side effects, what to do if experiencing side effects, and told about other methods. This paper looks at two measures of quality, one the MII and the other a constructed score (with six domains) using exploratory factor analysis with a large number of health facility variables. The two are examined in relation to a respondent’s time to discontinuation while in need over a 3-year period. The data are drawn from the URHI MLE surveys of facilities, women and clients, whereby aggregate measures of FPQ at facilities are linked to women, and client data are aggregated to represent FPQ at the facility level. The MII is put forth as an “actionable metric” for family planning (FP) quality. Among the EFA-based domains, information exchange, privacy/autonomy/dignity and technical competence were associated with significant reductions of discontinuation risk. Client satisfaction and Readiness for Choice on the other hand had significantly positive associations with discontinuation risk. 

1. Perhaps the most important chart in the paper is Figure 1, which traces the female sample from baseline through endline (discontinuation analysis of the calendar episodes). A similar chart regarding the SDPs (service delivery points) would have been helpful. 
2. The MII is measured as a proportion of the sample female population, who may or may not have all received modern contraceptive services.
3. The original sample for 5 sites is 13,140 households. The top box in Figure 1 begins with 5,217 women with matched baseline and endline interviews. Is the difference due to loss to follow-up and if so, what are the implication of selection bias for the analysis?
4. How was loss to follow-up (LFU) handled in the analysis with either the 1033 users for the MII model or the 938 users for the FPQ model? Did the authors just accept those who were relocated or try to weight for LFU?
5. In Figure 1, please double check the figures and also label the participants “users” (as opposed to “women”) from the second tier of boxes down to be consistent. You may also want to include a 1-2 sentence explanation of what a box plot is in Figure 2 (median and end points are shown).

 

 Some questions/comments about the modeling:

1. Did you include city in the models as fixed effects? Tables 2 and 4 suggests there are differences by city in quality score and client composition. Also the column title in Table 2 should be “Current FP users included in the analysis”. Be careful and consistent with the labeling.
2. What was the range of number of clients across the 124 high-volume facilities whose data were used to construct the facility-level MII measure? Table 2 appears to be discussed before Table 1. Given these are high-volume facilities, and not necessarily representative of all facilities in the 5 sites of Kenya, some caution is warranted with the results.
3. Tables 4-8 are discussed very cursorily. What is the rationale for the limited set of control variables (woman’s age, facility type and method type)? Why not also include other covariates shown in Table 1?
4. Table 4 by my count has 29 variables, not 38 variables used for the EFA (see the table title). The alpha values are by and large high, which is positive, but could be related to these 124 facilities being selectively high-volume. There is a word missing in the last variable “Provider…the client’s family planning preferences”.
5. Figure 3 is not very compelling about the relationship between the facility MII and the overall summative score. You have outlier values (facility MII=100) which are likely affecting the regression line fit. It and Table 7 could be moved to an appendix.
6. Temporality in establishing causal association needs to be clarified. The calendar covers a 5-year period; does exposure begin after the baseline round of data collection for female respondents and health facilities? Clarify that these measures precede the beginning of the calendar period. From Figure 1 it appears the 1009 episodes beginning 6 months after the woman’s baseline interview are excluded. If so, why? The description on bottom of pp. 4-5 is not mapped to the numbers in Figure 1.
7. The positive associations for the HRs for client satisfaction and readiness for choice warrant some discussion (Table 8).
8. After reading the limitations (pp. 14-15), one might ask what is the external validity of the findings in this paper? 

The paper is complex, with 8 tables and 3 figures, attempting to compare the sample retained versus analyzed, compile the calendar episodes, establish an EFA SFP score, then explain and contrast it with the MII (relationship graph) in both binary and ordinal forms, and then compare their performance with the discontinuation analysis. This is confounded by the need to follow the included and excluded females as well as track the health facilities retained. It’s a heroic attempt to analyze a rich set of data and tackle issues that are important to family planning research and evaluation. In some ways, it would have been best to first prepare the paper on the EFA of the FPQ and then use the score with the MII. 

 At a minimum, in addition to addressing as many of the above questions, I think the Conclusion should not sound so definitive. The first sentence should start out, “Within its limitations, this study…”. My other recommendation is going through the manuscript to correct labeling and ensure an outside reader can replicate the analytic steps for reproducibility purposes.

I confirm that I have read this submission and believe that I have an appropriate level of expertise to confirm that it is of an acceptable scientific standard, however I have significant reservations, as outlined above.

# Response

**Published:** 2020-01-03

**Authors:** Karla Feeser

**Affiliations:** Metrics for Management, USA

Thank you for your detailed review and attention. We have carefully considered your feedback and revised our manuscript accordingly. Please see our responses to each of your numbered comments below:

  

1. We have added more explicit information regarding the inclusion of SDPs in the analysis to the methodology of the paper.
2. We have added this clarification to paragraph 4 in the Introduction of the paper.
3. The response rate for longitudinal women’s data was 59% in Kenya – the lowest of the four MLE sites. Efforts were made by MLE researchers to mitigate this loss to follow-up; however, this level of attrition is to be expected in a complex urban setting such as this. 
4. This discontinuation models were not weighted for LFU. Among all participants, our analysis examined only current FP users at baseline, and the nature of our analysis (specifically, having to link women’s baseline episode to 5-year retrospective calendars collected at endline) require us to exclude many more. However, our main goals here were to not only look at associations between measures of quality and contraceptive discontinuation, but also to demonstrate how existing data and metrics can be used to explore pressing challenges in improving the quality of FP service delivery.
5. We have made the recommended changes to clarify and improve Figure 1 and Figure 2.

Modeling:

1. Thank you for the recommendation. We have made these changes to labelling in Table 2 for more consistency. City was not included in the model due to concerns that we would encounter a small cell problem.
2. We have added to the results section the range of number of clients across the 124 high-volume facilities whose data were used to construct the facility-level MII measure.
3. Individual characteristics at baseline – age, marital status, parity, education, wealth and method-type (long-term method or short-term method)— were considered as potential confounders of the relationship between quality and contraceptive use.  These characteristics may directly impact both a woman’s likelihood of continued contraceptive use and the way she is treated by providers and staff at a facility. Individual and facility type variables were screened for inclusion – final models were adjusted only for those covariates that were significant at p <0.1 when examined individually in a Cox proportional hazard model. This has been noted in the methodology section of the manuscript.
4. 38 variables were entered into EFA and variables were removed with each iteration. We have clarified this in the Results section, and removed “38” from the title of Table 4 to avoid confusion. Table 4 has been updated to reflect changes made to the overall analysis (specifically the switch from orthogonal to oblique rotation in the EFA), and all typos have been resolved.
5. We agree that Figure 3 does not add much to the overall narrative. It has been removed to alleviate some of the complexity of the paper.
6. For some women, data on facility quality were collected up to a year after the baseline data on current contraceptive use were collected. However, because we don’t expect that quality changed very much during that time, we chose not to exclude women whose reported baseline episodes may have begun before SDP assessments occurred. This has been added to the limitation sections, although we do not believe that this would significantly impact our findings. Women’s baseline episodes, as reported in baseline interviews, were identified in the five-year calendar collected in endline interviews by matching on the month and year of the episode start date and on method type, as close as possible. Given the imperfect recall expected with data of this nature, we allowed for a 6-month margin of error on method start date. Because all women included in the analysis were current FP users at baseline, we were able to immediately exclude any episode identified in the 5-year retrospective calendar that began more than 6 months after the baseline interview.
7. We agree that the finding that increased client satisfaction was associated with discontinuation is strange. We’ve added a stratified analysis to examine the possibility of effect modification by provider-controlled versus woman-controlled methods. The results are displayed in Appendix A. The finding holds among women who are *not *using provider-controlled methods, but is no longer significant among women who are using provider-controlled methods. Although our factor analysis process grouped the items in this domain together, other frameworks include these measures in different conceptual areas. For example, Jain (2019) would  not consider wait time an indication of process quality, while Hutchison et al (2011) defined client satisfaction very broadly, encompassing aspects of our domains of client satisfaction, privacy and comfort, and information provision. Given that other studies have found a conceptual and mathematical relationships between client reported measures and observed quality, this particular finding is not well explained within the present study. This has been noted in the limitation section of the discussion. The positive associations for the HRs for the “Readiness for Choice & Management Support” domain are not significant at a threshold of p < 0.05, and have wide confidence intervals that range from below to above 1.0 in both the unadjusted and adjusted models. So, we consider this finding to be indicative of no association in either direction.
8. We agree that our analysis has many limitations and have clarified our goals throughout the body of the manuscript. Specifically, we note that one key goal of the paper was to demonstrate how existing metrics and data can be used to assess and improve quality of care related to family planning health outcomes through secondary analyses such as these.

# Peer review: Reviewer response for version 1

**DOI:** 10.21956/gatesopenres.14081.r27119

**Published:** 2019-06-10

**Authors:** Kelsey Holt

**Affiliations:** 1 Department of Family and Community Medicine, University of California, San Francisco, San Francisco, CA, USA

Thank you very much for the opportunity to review this submission. In the article, the authors present results of an analysis of data from household surveys and facility assessments related to the link between quality of care and contraceptive continuation. Though much research exists analyzing a similar question, the article is unique in utilizing the MII as an exposure variable. This is important because the MII is increasingly being used as a standardized quality indicator of client experience and it is important to know how it relates to contraceptive continuation. I have detailed a number of substantive recommendations to strengthen the paper.

1. I appreciate the use of “discontinuation-while-in-need” as an innovative way to describe the outcome of interest and signal that not all discontinuation is created equal. Please be sure that 1) this is evenly used throughout (e.g., in the Conclusions it is not used); and 2) that when it is first used the definition is given so that it is clear that it does not include people who have switched to another method. If possible, this clarification should also be added to the Introduction of the Abstract (i.e., “discontinuation-while-in-need *without switching*”) as this will help normalize switching as not being a negative outcome. Also on page 4 where the DHS data from Kenya is reported, it would be important to clarify whether the 31% discontinuation figure allowed for switching.
2. Also related to use of discontinuation-while-in-need as an outcome, I suggest more justification in the body of the paper (and abstract if at all possible) as to why this is an important outcome. In the first paragraph of the Introduction, a circular argument is given (quality is associated with continuation which therefore means that continuation is an important outcome? But why?) Perhaps the authors want to justify this by pointing to the fact that a person’s ability to use contraception when they don’t want to be pregnant is an important outcome because it is an indication that the health system has met their needs/right to reproductive autonomy, either by helping them identify (and making available) a method that will work for them or by imbuing in them the confidence to return for a different method if the first doesn’t work out.
3. In the 4 ^th^ paragraph of the Introduction, the description of the MII as distinct from tools derived from Bruce, and use of the word “but” before “measures it from the client perspective,” is confusing. Please clarify. My understanding is that the MII was created based on selection of questions that already existed from the Bruce framework (and that were already standard in the DHS, etc.), and thus the MII in many ways is a continuation of this long tradition.
4. The first sentence of the 5 ^th^ paragraph (“the MII captures information exchange during the counseling session and a woman’s understanding of having received that information, giving it the potential to approximate the overall quality of FP services.”) is unclear – why does the information exchange focus inherently suggest that this would be a sufficient overall quality measure? Please clarify or reword.
5. The other point made further down in paragraph 5 of the Introduction about the importance of placing client perspectives at the center of quality measurement is important and could be elaborated on, for example by linking with new quality frameworks from the WHO and the Lancet Commission for High Quality Health Systems which emphasize the importance of client experience as important in and of itself (regardless of external observations of how care is delivered).
6. Re: the description on page 4 of the facility data collection (beginning “the present study is a secondary analysis…”): it was confusing on first read how the “modified version of the QIQ” relates to the description in the abstract and later in the paper of this facility data collection as a “comprehensive service delivery point assessment.” Standardizing the language would help with ease of understanding for the reader when this facility data collection is described.
7. In the last sentence of the Introduction, the authors state that part of their objective was to “explore the feasibility of these measures in the context [of] quality monitoring at the service delivery point.” However, no data on feasibility of measure implementation is provided in the paper. Please clarify/rephrase.
8. Inclusion criteria: it is not clear to me why the 96 women without information on where they received their method were excluded from the MII analyses? Please consider including them or justify why they were left out even though location of service delivery was not essential to the analysis.
9. Model selection: when describing covariates included in the models, it would be important to add further detail as to conceptually why these variables were selected. Presumably individual characteristics such as age, parity, etc., were conceptualized as potential confounders of the relationship between quality and contraceptive use (e.g., if younger people are both treated differently by providers and also inherently more likely to be less consistent in contraceptive use?). For facility type and method type, why were these included? Was effect modification considered and if so why were no interaction analyses conducted?
10. In the description of the facility quality variables, more detail on the selection of a single representative provider is justified – why only one? Why choose the likely highest performers?
11. In descriptive Tables where statistical tests were used to examine the possibility of statistically significant differences between sub-groups were performed, consider adding the p-values and a description of the approach taken (Chi-Square/T-test?), depending on journal convention.
12. Justification for use of orthogonal rotation to produce uncorrelated factors is needed; why did the authors believe that dimensions of quality would be unrelated to each other? One might imagine that some elements of quality, particularly those that touch on client-provider interactions, would be related. And, if uncorrelated factors are assumed, how do the authors then justify a composite quality score combining the sub-scale scores? Consider switching to oblique rotation or justifying the choice of orthogonal rotation, and please also clarify in the Discussion whether authors believe there is justification for combining sub-scale facility quality scores into composite scores.
13. The Discussion section requires more engagement with prior literature that has used similar approaches to linking facility level quality data to household survey contraceptive use data; notably a recent study by Tumlinson *et al.* (2015 ^1^) reported on a very similar analysis, also in urban Kenya. How are these findings similar or different to what others have found related to facility level quality and household contraceptive use? (For example, Fruhauf *et al.*, 2018 ^2^; Do and Koenig, 2007 ^3^; Arends-Kuenning and Kessy, 2007 ^4^; Hong *et al.*, 2006 ^5^; Ketende *et al.*, 2003 ^6^; Feyisetan and Ainsworth, 1996 ^7^).
14. Table 4: it would be very informative to indicate the data source for each of the variables (i.e., exit interview, versus observation, versus provider interview); this will help the reader interpret the data.
15. Many of the factor loadings are below 0.4, and most of the Cronbach alphas are below 0.7 or even below 0.5), suggesting weak scales. The Limitations section should include acknowledgement of this.
16. Relatedly, justification should be provided for not considering content validity when selecting the sub-scales from EFA; standard practice in scale development is to weigh content validity and psychometric data when selecting items. As mentioned in the Limitations section, the scales are limited in this way, but it is unclear why the authors did not do it differently.
17. Labelling the 4 ^th^ domain “privacy, autonomy, and dignity,” feels misplaced given that 2/3 items are related to privacy. The third is related to comfort asking questions and it is not clear how that item reflects “autonomy” or “dignity”; I suggest a more modest description of this domain to more accurately represent what it covers (e.g., “counseling environment” or “privacy and ease with provider”? wording needs help…)?
18. There is a typo in the last item of Table 4.
19. Table 5: It would be helpful to indicate somewhere what the possible score ranges were for each domain (ranges are given but presumably the full range of possible scores were not always reported?).
20. Table 7 – it is confusing to have different reference groups for the two different MII models. It would greatly aid interpretation to be consistent.
21. It is perplexing that there is no mention of a human rights-based approach to service delivery, given the paradigm shift that has been taking place in recent years in the family planning field. Even on page 14 when the authors highlight the importance of privacy and autonomy, they surprisingly don’t link this to a human rights frame. I recommend making the explicit connection with a rights-based approach to FP service provision in the Intro and Discussion sections.
22. The finding that increased client satisfaction was associated with discontinuation (including a very large OR) deserves more attention. The authors attempt to explain the finding by saying that content validity may be low; however, other than wait time, the other three variables are very clearly about provider treatment, making it difficult to imagine that the construct is measuring something very qualitatively different. Also, explaining this finding by saying that client-report is inherently unreliable appears to go directly against the authors conclusions that the MII is a good measure of quality, and also the idea that client report of their experience is inherently valuable even if it is different from “objective” observations. Even given courtesy bias, why would there be such a strong relationship in the “wrong” direction? Finally, is it possible that some women continue contraception, particularly long-acting methods, due to pressure/coercion from providers and therefore poorer interactions could be associated with inability to remove? A stratified analysis to examine the possibility of effect modification by provider-controlled versus woman-controlled methods is advisable to explore this possibility.

I confirm that I have read this submission and believe that I have an appropriate level of expertise to confirm that it is of an acceptable scientific standard, however I have significant reservations, as outlined above.

## References

[1] : Quality of Care and Contraceptive Use in Urban Kenya. *Int Perspect Sex Reprod Health*.2015;41(2) : 10.1363/4106915 69-79 10.1363/4106915 26308259PMC4548971

[2] : Measuring family planning quality and its link with contraceptive use in public facilities in Burkina Faso, Ethiopia, Kenya and Uganda. *Health Policy Plan*.2018;33(7) : 10.1093/heapol/czy058 828-839 10.1093/heapol/czy058 30010860PMC6097453

[3] : Effect of family planning services on modern contraceptive method continuation in Vietnam. *J Biosoc Sci*.2007;39(2) : 10.1017/S0021932006001453 201-20 10.1017/S0021932006001453 16817990

[4] : The impact of demand factors, quality of care and access to facilities on contraceptive use in Tanzania. *J Biosoc Sci*.2007;39(1) : 10.1017/S0021932005001045 1-26 10.1017/S0021932005001045 16359581

[5] : Family planning services quality as a determinant of use of IUD in Egypt. *BMC Health Serv Res*.2006;6: 10.1186/1472-6963-6-79 79 10.1186/1472-6963-6-79 16792810PMC1553443

[6] : Facility-level reproductive health interventions and contraceptive use in Uganda. *Int Fam Plan Perspect*.2003;29(3) : 10.1363/ifpp.29.130.03 130-7 10.1363/ifpp.29.130.03 14519590

[7] : Contraceptive Use and the Quality, Price, and Availability of Family Planning in Nigeria. *The World Bank Economic Review*.1996;10(1) : 10.1093/wber/10.1.159 159-187 10.1093/wber/10.1.159

# Response

**Published:** 2020-01-03

**Authors:** Karla Feeser

**Affiliations:** Metrics for Management, USA

Thank you for your detailed review and attention. We have carefully considered your feedback and revised our manuscript accordingly. Please see our responses to each of your numbered comments below:

  

1. We agree that “discontinuation-while-in-need” provides an important distinction from all forms of discontinuation in our outcome of interest, and that switching to another method need not be interpreted as a negative outcome, given that the users are still using contraception while in need. We have standardized our language from “discontinuation” to “discontinuation-while-in-need” throughout the manuscript, and clarified the definition to “discontinuation-while-in-need without switching” when it is first introduced. Regarding the DHS data from Kenya reported on page 4, the 31% of episodes that were discontinued does include switching (11% of episodes). This has been clarified in the body of the manuscript.
2. We apologize for our lack of clarity in our justification for discontinuation-while-in-need as an outcome in this analysis. What we intended to say was that it has been demonstrated elsewhere that the quality of services can impact continued use of contraception and that this provides a good basis for its use in our models from an analytical perspective. Additionally, we aimed to identify a need: We know that these two things (quality and discontinuation) are associated, and can reasonably expect improvements in quality to lead to reductions in discontinuation rates, but we haven’t necessarily identified with any certainty which aspects of quality are truly related nor do we have a good way to measure process and structural quality in real time. We agree that there is an additional point to make here – specifically, that discontinuation-while-in-need is an important outcome in and of itself, for exactly the reasons you have stated here. We have added this justification to the introduction of the manuscript. 
3. It is true that the MII is in many ways a continuation of the Bruce Framework. We have clarified that the MII incorporates only one-aspect of the Bruce Framework (information provision), compared to the more comprehensive assessment tools referenced in the preceding paragraph.
4. We have reworded this sentence to be more clear.
5. We have added a brief of discussion of these new quality frameworks from the WHO, the U.S. National Academy of Medicine and the Lancet Commission on High Quality Health Systems to paragraph five.
6. Thank you for the recommendation. In paragraph 2, we describe the QIQ as an example of a comprehensive service delivery assessment guided by the Bruce framework. Our hope is that this will provide clarity for the reader.
7. We agree that this was phrased ambitiously and have removed this claim from this sentence.
8. Although MII was measured at the woman-level, the MII to Discontinuation model was adjusted for Facility Type. These 96 women would have been dropped from the model for that reason. We have added this explanation to the methods section.
9. Individual characteristics at baseline – age, marital status, parity, education, wealth and method-type (long-term method or short-term method)— were considered as potential confounders of the relationship between quality and contraceptive use.  These characteristics may directly impact both a woman’s likelihood of continued contraceptive use and the way she is treated by providers and staff at a facility. Individual and facility type variables were screened for inclusion – final models were adjusted only for those covariates that were significant at p <0.1 when examined individually in a Cox proportional hazard model.
10. We chose to select one representative provider rather than averaging the values from all provider surveys and losing detail in the responses. We used criteria to identify those who were likely to be most knowledgeable of facility operations, though we do recognize that these provider’s may also be higher performers.
11. Thank you for the recommendation. P-values have been added to Table 1 corresponding to Chi-squared tests for independence.
12. Per your recommendation we have chosen to update the EFA to use oblique rotation instead of orthogonal rotation. This change to our EFA did not impact the number or overall identity of the resulting factors; however, some variables were ultimately assigned to different factors, which changed the resulting quality domain scores for each category in the discontinuation models.  The methods, results and corresponding tables in the manuscript have all been updated. Regarding the use of a composite score, we chose to remove our presentation of an overall score from the manuscript all together. There are many different reasons and ways to calculate and combine quality domains into a composite score, but that was not the goal of this analysis. Furthermore, we felt that removing references to an overall quality score would alleviate some of the complexity in the manuscript.
13. We have added further engagement with the literature to the discussion section, and are grateful for the recommended sources.
14. We appreciate this recommendation and have added a column to the table detailing the 25 variables retained throughout EFA that indicates the data source for each variable.
15. We have acknowledged this limitation of our analysis in the discussion section of the paper.
16. Through the course of updating our EFA w/ oblique rotation, we seized the opportunity to consider content validity more mindfully alongside the psychometric data as we assigned variables to each factor. The methods have been updated to reflect this.
17. We have changed the named of the “Privacy, autonomy and dignity” domain to “Privacy & Comfort”.
18. Table 4 has been replaced to reflect the new EFA results, and the typo has been removed.
19. Possible score ranges have been added to the column headers in Table 5.
20. Thank you for the recommendation. We have elected to keep Table 7 as is, keeping ‘0’ as the reference group for both variables.
21. We agree that the human rights-based approach to service delivery is an important perspective, although not the focus our paper. We have added a brief link to the rights-based approach to FP service provision to the discussion.
22. We agree that the finding that increased client satisfaction was associated with discontinuation is strange. We have added a stratified analysis to examine the possibility of effect modification by provider-controlled versus woman-controlled methods. The results are displayed in Appendix A. The finding holds among women who are *not *using provider-controlled methods, but is no longer significant among women who are using provider-controlled methods. Although our factor analysis process grouped the items in this domain together, other frameworks include these measures in different conceptual areas. For example, Jain (2019) would  not consider wait time an indication of process quality, while Hutchison et al (2011) defined client satisfaction very broadly, encompassing aspects of our domains of client satisfaction, privacy and comfort, and information provision. Given that other studies have found a conceptual and mathematical relationships between client reported measures and observed quality, this particular finding is not well explained within the present study. This has been noted in the limitation section of the discussion.
